# Transvaginal Sonography: perception and attitude of Nigerian women

**DOI:** 10.1186/s12905-017-0413-z

**Published:** 2017-07-27

**Authors:** Mark C Okeji, Kennedy K Agwuna, Chika N Ihudiebube-Splendor, Iliyasu Y Izge, Kelechi K Ekuma, Jennifer O Emeter

**Affiliations:** 1 Department of Medical Radiography and Radiological Sciences, Faculty of Health Sciences and Technology, University of Nigeria, Enugu Campus, Enugu, Enugu State Nigeria; 2 Department of Radiation Medicine, Faculty of Medicine, College of Medicine, University of Nigeria Enugu Campus, Enugu, Enugu State Nigeria; 3 Department of Nursing Sciences, Faculty of Health Sciences and Technology, University of Nigeria, Enugu Campus, Enugu, Enugu State Nigeria; 4 Department of Radiography, Faculty of Clinical Science, College of Health Sciences, Usmanu Danfodiyo University, Sokoto, Sokoto State Nigeria

**Keywords:** Transvaginal sonography, Attitude, Perception, Women, Nigeria

## Abstract

**Background:**

To assess the attitude to and perception of transvaginal sonography (TVS) among Nigerian women of mixed educational status in order to ascertain factors that may prevent them from submitting to TVS when recommended.

**Methods:**

A Cross-sectional survey was adopted for the study. In all, one missionary, one government and eight private hospitals were enlisted. The instruments for data collection were visual analogue scale (VAS), to ascertain patients’ pain/discomfort experience, and a researcher-developed semi-structured questionnaire. The level of pain/discomfort on the VAS was categorized into four on a scale of 100. The categories were: 0–5 (no pain), 6–40 (mild pain), 41–74 (moderate pain), and 75–100 (severe pain).

**Results:**

Majority (50.6%) of the respondents who attained secondary education had positive attitude to TVS. Also majority of the respondents (63.1%) preferred female sonographers. Majority of the respondents (54.1%) perceived TVS as not embarrassing, 78% did not consider it stressful, 96.9% reported that the sonographers were professional, 46.7% felt that a chaperon was needed, 98.4% reported there were enough privacy and 84.7% reported they needed prior information. Most of the respondents (82%) were willing to consent to TVS in future, 90.5% reported no pain, 8.6% reported mild pain/discomfort and 0.9% reported moderate pain.

**Conclusions:**

Majority of our respondents had positive attitude to TVS and were willing to consent to TVS in future, hence it was acceptable to them. It was however observed that acceptability increased with increasing academic status.

## Background

Transvaginal sonography (TVS) is a diagnostic tool for the evaluation of the female pelvis and involves the use of high frequency transducer placed in the vagina where it is in close anatomic proximity to the pelvic structures. It is ideal for the assessment of ovulation [1] and in oocyte recovery for the management of infertile patients [2]. The procedure overcomes the difficulties encountered in imaging obese patients, patients with large amount of bowel gas, and those with inadequate bladder filling [3]. Since Transvaginal sonography invades the privacy of the female patients, there is need for a study that will focus on the attitude to and perception of the procedure by female patients. Several studies had reported its acceptability and not being embarrasing [4–8]. However a study by Onderi et al. [9] reported that it was embarrassing to majority of the patients he studied. Most of these studies conducted so far in Nigeria and other countries were on literate population. We therefore sought to ascertain the attitude to and perception of TVS by women of diverse tribes most of who engaged in buying and selling in Onitsha, Anambra State, Nigeria. We also assessed patients’ experience of pain and choice of sex of the sonographer.

## Methods

This study adopted the cross-sectional survey design. Ten hospitals that incorporate transvaginal sonography for obstetrics and gynaecological cases were purposively enlisted into the study. In all, one missionary hospital, one government hospital and eight private hospitals were enlisted. All the 255 patients who consented to participate in the study were scanned by qualified sonographers with TVS, within the period of the study. The procedure was explained to the patients before the commencement of the examination. The instruments for data collection were visual analogue scale (VAS), to ascertain patients’ pain/discomfort experience, and a researcher-developed semi-structured questionnaire divided into three sections A, B and C. Sections A elicited information on some demographic variables while section B sought data on the knowledge of TVS. Section C sought data on attitude to and perception of TVS among others. The researcher-developed questionnaire was validated by three experts from Department of Medical Radiography and Radiological Sciences of University of Nigeria. The instrument was pilot tested in Enugu State and its reliability computed using Cronbach alpha which gave a coefficient of 0.81. Data generated were subjected to descriptive statistics and analyzed using Chi square and Pearson product moment correlation. Probability value (*p*< 0.05) was considered statistically significant.

## Results

All the administered 255 VAS and semi-structured questionnaires were completed and returned giving a return rate of 100%. Majority (47.1%) of the respondents were within the age group of 26 to 35 years, 68.2% were married and 55.3% had secondary school education. Tertiary education group includes those who acquired post-secondary school education in accredited institutions and they constitute 38.8% of the respondents (Table 1). Majority (50.6%) of the respondents who attained secondary education had positive attitude to TVS. Also majority of the respondents (63.1%) preferred female sonographers (Table 2).

**Table 1:** Demographic characteristics of the respondents (*n* = 255)

Characteristics	Frequency (%)
Age of respondents (years)	
15–25	59 (23.1%)
26–35	120 (47.1%)
36–45	58 (22.7%)
> 45	18 (7.1%)
Marital status	
Single	69 (27.1%)
Married	174 (68.2%)
Divorced	5 (2.0%)
Widow	7 (2.7%)
Educational status	
Primary	9 (3.5%)
Secondary	147 (57.7%)
Tertiary	99 (38.8%)

**Table 2:** Attitude of the women based on educational status and choice of sonographer

Characteristics	Positive	Negative	Indifferent
Primary	5 (2%)	4 (1.6%)	0
Secondary	129 (50.6%)	16 (6.3%)	2 (0.8%)
Tertiary	93 (36.5%)	2 (0.8%)	4 (1.6%)
Total	227 (89%)	22 (8.6%)	6 (2.4%)
Preferred female sonographer	161 (63.1%)	67 (26.3%)	27 (10.6%)

Majority of the respondents (54.1%) considered TVS not embarrassing, 78% did not consider it stressful, 96.9% felt that the sonographers were professional, 46.7% felt that a chaperon was needed, 98.4% reported there were enough privacy and 84.7% reported they needed prior information (Table 3).

**Table 3:** Perception of TVS by the respondents

Perception	Yes (%)	No (%)
Embarrassing	117 (45.9%)	138 (54.1%)
Stressful	53 (21.2%)	202 (78.8%)
Sonographer was professional	247 (96.9%)	8 (3.1%)
Need for a chaperon	119 (46.7%)	136 (53.3%)
There was enough privacy	251 (98.4%)	4 (1.6%)
Prior information was adequate	216 (84.7%)	39 (15.3%)
Will consent to TVS in future	209 (82%)	46 (18%)

To assess the pain/discomfort experienced by the respondents, the visual analogue scale (VAS) was used. This scale had been used in previous studies to assess pain/discomfort [10, 11]. The participants were asked to mark the level of pain/discomfort on the VAS. The level of pain/discomfort was categorized into four on a scale of 100. The categories were: 0–5 (no pain), 6–40 (mild pain), 41–74 (moderate pain), and 75–100 (severe pain).

**Fig. 1:**
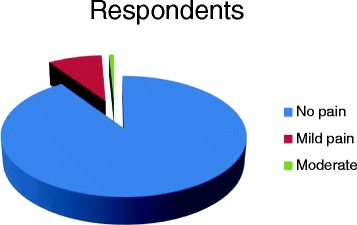
Respondents perception of pain/discomfort on the VAS

Most of the respondents (90.5%) reported no pain, 8.6% reported mild pain/discomfort and 0.9% reported moderate pain (Fig. 1).

## Discussion

Our study revealed that majority of the respondents (47.1%) were within the age range of 26 to 35, married (68.2%) and attained secondary school education (57.7%). The attitude to TVS by majority (89%) of the respondents was positive. Positive attitude was positively and significantly related to the increased level of education (*r* = 0.69). Majority of the respondents (63.1%) had positive preference for female sonographers and 10.6% were indifferent. This is similar to some previous studies [12, 13] where the respondents reported preference for female sonographers. Few of the respondents (26.3%) had negative preference for female sonographers which they attributed to; males being more skilful, being used to male obstetricians and gynaecologists. Majority of the respondents (54.1%) did not perceive TVS as embarrassing while 45.9% of the respondents felt it was embarrassing. However there was no significant difference (*p* > 0.05) between the respondents who felt embarrassed and those who did not feel embarrassed. Our finding was contrary to a study in Kenya [9] where they found majority of the respondent reporting being embarrassed. The percentage of respondents in our study who felt embarrassed was study [14] where only 5.2% of the respondents reported feeling embarrassed during the scan. The higher percentage of respondents in our study who felt embarrassed may be attributed to the higher number of respondents (84.7%) who reported that prior information was necessary before commencement of the scan. Two hundred and two respondents (78.8%) perceived TVS as non stressful while 209 (82%) reported that they will consent to TVS in future, implying that it is acceptable to them. Two hundred and forty-seven respondents (96.9%) reported that the sonographers were professional and 98.4% reported that enough privacy was accorded them. This finding is similar to a previous study in UK [14] where 97.7% and 93.3% of the women reported that they were handled professionally and accorded enough privacy. However 46.7% of the respondents reported the need for a chaperon. The respondents who desired a janitor were mostly primips.

On the respondents’ assessment of pain/discomfort, 90.5% reported no pain/discomfort, 8.6% reported mild pain/discomfort while 0.9% reported moderate pain/discomfort. Some previous studies had also reported TVS to be associated with pain/discomfort at varying levels [14–17]. The following factors were presented in literature as being related to pain experience; age, hysterectomy, experience/skill of the sonographer and prolonged scanning time [14, 18]. However our study revealed that pain was more in primips and in respondents with lower educational status.

One of the limitations of our study was not randomizing the respondents based on prior explanation of the procedure or not for the assessment of pain. Also environmental factors and the responses of the participants scanned by male/female sonographers were not compared in the pain assessment.

## Conclusion

Majority of our respondents had positive attitude to TVS and also willing to undergo the investigation in future, hence it was acceptable to them. It was however observed that acceptability increased with increasing academic status. Few respondents reported mild/moderate pain/discomfort. We recommend prior information, provision of calm and conducive environment and employment of skilful/experienced female sonographers as measures to reduce embarrassment and perceived pain during TVS.

## Abbreviations

TVS: Transvaginal sonography
VAS: Visual analogue score
